# Microglial CD206 Gene Has Potential as a State Marker of Bipolar Disorder

**DOI:** 10.3389/fimmu.2016.00676

**Published:** 2017-01-09

**Authors:** Masahiro Ohgidani, Takahiro A. Kato, Yoshinori Haraguchi, Toshio Matsushima, Yoshito Mizoguchi, Toru Murakawa-Hirachi, Noriaki Sagata, Akira Monji, Shigenobu Kanba

**Affiliations:** ^1^Department of Neuropsychiatry, Graduate School of Medical Sciences, Kyushu University, Fukuoka, Japan; ^2^Innovation Center for Medical Redox Navigation, Kyushu University, Fukuoka, Japan; ^3^Department of Psychiatry, Graduate School of Medical Sciences, Saga University, Saga, Japan

**Keywords:** bipolar disorder, rapid cycling, microglia, CD206, induced microglia-like (iMG) cells, state marker, M1/M2 polarization, translational research

## Abstract

The pathophysiology of bipolar disorder, especially the underlying mechanisms of the bipolarity between manic and depressive states, has yet to be clarified. Microglia, immune cells in the brain, play important roles in the process of brain inflammation, and recent positron emission tomography studies have indicated microglial overactivation in the brain of patients with bipolar disorder. We have recently developed a technique to induced microglia-like (iMG) cells from peripheral blood (monocytes). We introduce a novel translational approach focusing on bipolar disorder using this iMG technique. We hypothesize that immunological conditional changes in microglia may contribute to the shift between manic and depressive states, and thus we herein analyzed gene profiling patterns of iMG cells from three patients with rapid cycling bipolar disorder during both manic and depressive states, respectively. We revealed that the gene profiling patterns are different between manic and depressive states. The profiling pattern of case 1 showed that M1 microglia is dominant in the manic state compared to the depressive state. However, the patterns of cases 2 and 3 were not consistent with the pattern of case 1. CD206, a mannose receptor known as a typical M2 marker, was significantly downregulated in the manic state among all three patients. This is the first report to indicate the importance of shifting microglial M1/M2 characteristics, especially the CD206 gene expression pattern between depressive and manic states. Further translational studies are needed to dig up the microglial roles in the underlying biological mechanisms of bipolar disorder.

## Bipolar Disorder and Microglia

The pathophysiology of bipolar disorder has yet to be well understood, while recent studies have indicated abnormal immunological functions may be a contributing factor (1, 2). Microglia, immune cells in the brain, play important roles in the process of brain inflammation, and recent positron emission tomography (PET) studies have shown microglial overactivation in the brain of patients with various psychiatric disorders including bipolar disorder (3–8). Based on the above evidence, microglia has been highlighted in the study of various psychiatric disorders to understand the underlying biological mechanisms (9–12).

We have recently developed a technique to induced microglia-like (iMG) cells from peripheral blood (13) and are now confirming the utilities of this technique for psychiatric research (14, 15). The underlying mechanisms of the bipolarity between manic and depressive states have yet to be clarified. Immunological conditional changes in microglia may contribute to the manic–depressive shift in bipolar disorder. In the field of immunology, M1/M2 polarization is recognized as a useful marker of macrophages and related cells including microglia. Polarization pattern is well known to distinguish functional phenotypes: pro-inflammation (M1) and anti-inflammation (M2) (16, 17). Recently, M1/M2 polarization has been highlighted in the understanding of psychiatric disorders (18–20). However, there is no research analyzing M1/M2 polarization of microglia in patients with bipolar disorder. We hypothesize that the expression profile of inflammation-related genes known as M1 (CD45, CD80, HLA-DR, TNF-α, IL-1β, and IL-23) or M2 markers (CD206, CD209, CD23, BDNF, IL-10, and CCL18) of microglia may shift between manic and depressive states. In order to clarify this hypothesis, we herein analyzed iMG cells from three patients with rapid cycling bipolar disorder during both manic and depressive states, respectively.

## M1/M2 Microglia and Bipolar Disorder

**Figure 1:**
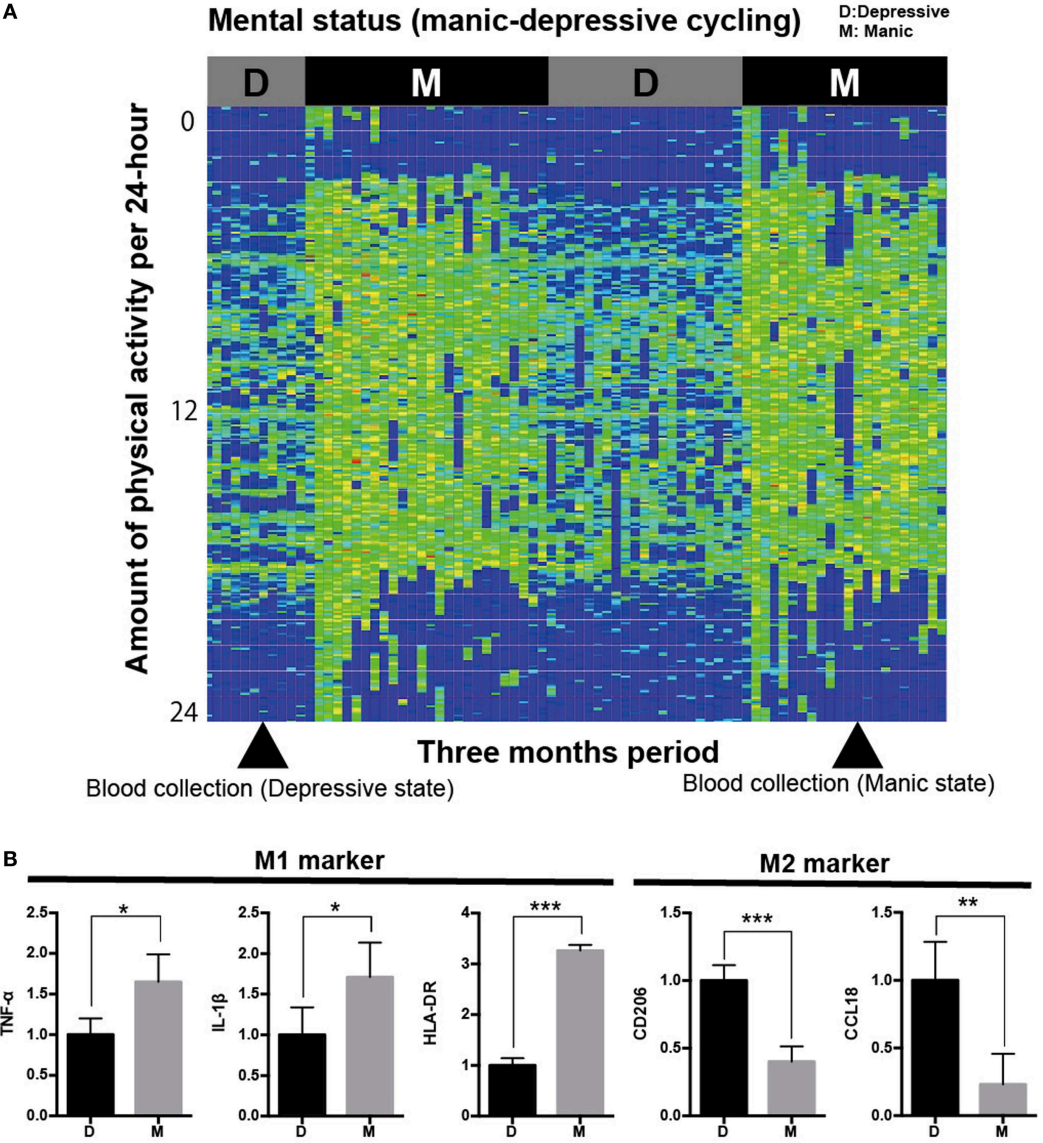
**Analysis of iMG cells from a typical case with rapid cycling bipolar disorder**. **(A)** Physical/mental activity of a patient with rapid cycling bipolar disorder for 3 months (case 1). **(B)** Gene profiling pattern of iMG cells from case 1 showed that M1 microglia is dominant in the manic state compared to the depressive state.

**Figure 2:**
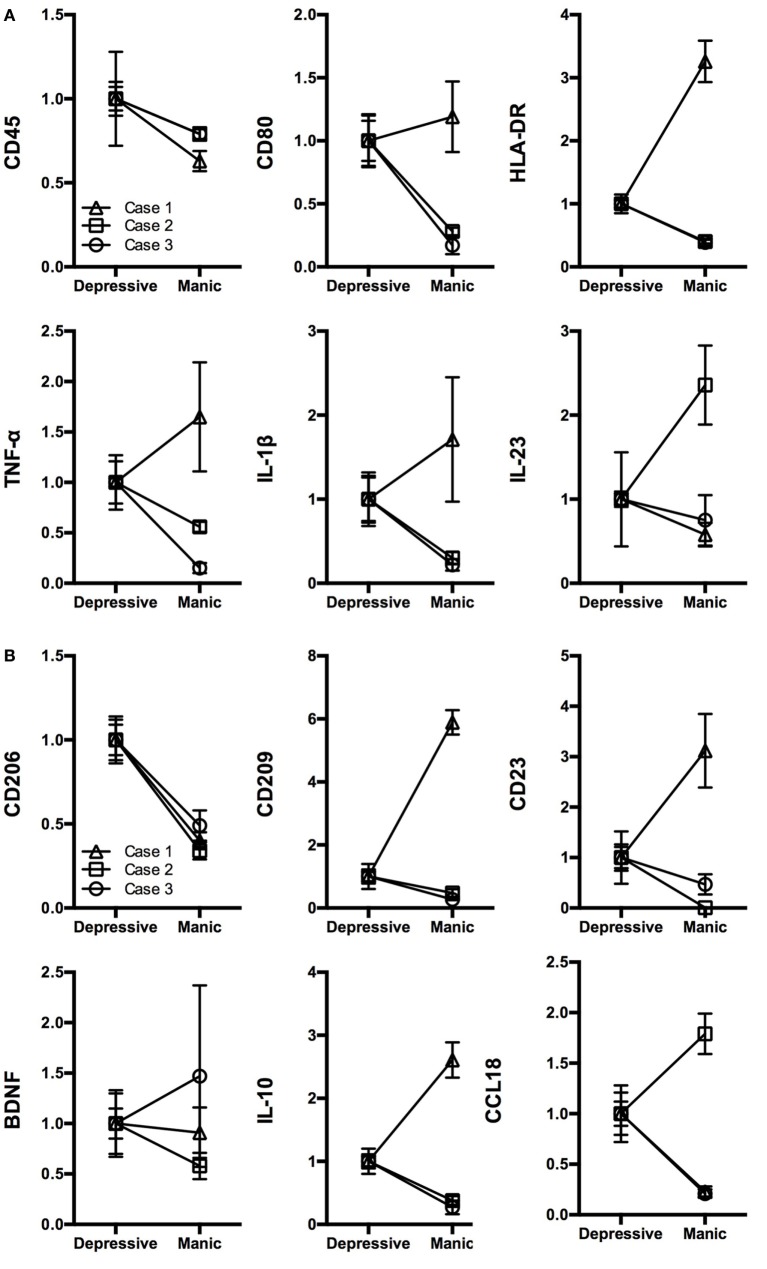

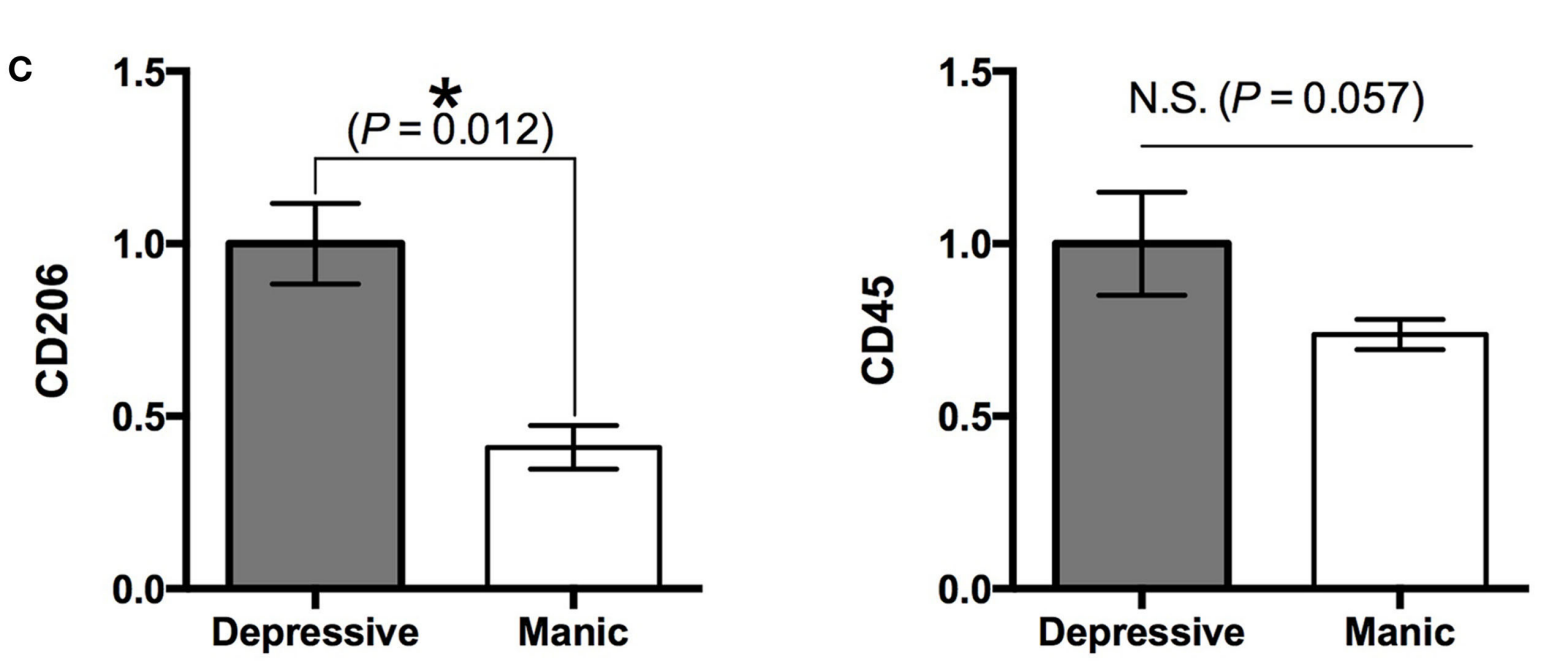
**Gene profiling pattern of iMG cells between depressive and manic states among three patients. (A)** M1 markers, **(B)** M2 markers, and **(C)** statistical analysis of CD206 and CD45 among the three patients (mean value ± SD).

Patients’ demographic data are shown in Table S1 in Supplementary Material. We produced iMG cells of each patient from both manic and depressive states and compared the gene expression profiles between both states. Relative gene expression (normalized by depressive state) of M1 and M2 markers are shown in Figures 1 and 2A,B, respectively.

We revealed that the gene profiling patterns of iMG cells are different between manic and depressive states. The profiling pattern of case 1 showed that M1 microglia is dominant in the manic state compared to the depressive state (Figure 1B). However, the patterns of cases 2 and 3 were not consistent with the pattern of case 1 (Figures 2A,B).

For M1 markers, CD45 was downregulated in the manic state among all three patients. Other M1 markers such as TNF-α, IL-1β, IL-23, CD80, and HLA-DR shifted differently among the patients (Figure 2A). For M2 markers, CD206 was downregulated in the manic state among all three patients. Other M2 markers such as BDNF, IL-10, CCL18, CD23, and CD209 shifted differently among the patients (Figure 2B). Thus, we performed statistical analysis [Student’s *t*-test (two-tailed)] among the three patients. As shown in Figure 2C, CD206 was significantly downregulated in the manic state (*p* = 0.012). On the other hand, CD45 showed no significant difference between manic and depressive states (*p* = 0.057).

## CD206 and Microglia

In the present study, we have shown that downregulation of the CD206 gene of iMG cells in the manic state was consistent across all three patients with bipolar disorder. CD206, known as a mannose receptor, is a 175-kDa transmembrane protein, mostly expressed by macrophages, dendritic cells, and endothelial cells. This receptor selectively and efficiently captures mannosylated ligands such as microbial antigen (21). In the brain, CD206 is also expressed in microglia (22, 23) and astrocytes (24, 25). CD206 is widely recognized as a typical M2 microglial marker (23, 26, 27). CD206 has some important cellular functions especially in pinocytosis and phagocytosis on microglia (22, 25, 28–30). Therefore, CD206 may be critical as the first step in the recognition and capture of pathogens in the brain (25). To date, there are no studies focusing on microglial CD206 in psychiatric disorders including bipolar disorder. In the present study, CD206 was downregulated in the manic state. This finding might suggest that the manic state of microglia is more vulnerable to the pathogen and/or insoluble matter compared to the depressive state. Based on the present results, M2 microglia may be dominant in the depressive state in patients with bipolar disorder, especially rapid cycling patients. Further translational investigations should be conducted to clarify our hypothesis.

## Limitation and Future Perspectives

One major limitation of the present study is that all three patients were on medication. Given that it took 2 weeks to induce iMG cells, the influence of medication is assumed to be minimal. Additional studies are needed in medication-free patients. On the other hand, recent PET studies have suggested microglial overactivation in patients with bipolar disorder (7, 8). Thus, human PET studies should be conducted in patients with bipolar disorder during both manic–depressive states. However, even the most advanced brain imaging techniques cannot analyze M1/M2 polarization in the human brain, and thus we believe that the iMG technique has an advantage for such analyses in translational research.

A recent study has revealed that the origin of brain microglia is primitive macrophages migrated from the yolk sac before embryonic day 8 (31). One of the limitations in the present study is that iMG cells are not actual microglial cells in the brain. However, we believe that our iMG cells are surrogate cells, which can represent some of the characteristics of brain microglial cells (13). Further comparison studies are needed to investigate the similarity and differences between brain microglial cells and iMG cells in more detail.

The life span of intravascular blood monocytes is only a few days long (32). Thus, we believe that the iMG cells from peripheral blood monocytes are useful not only as a trait marker but also as a state marker in order to assess a variety of mental states. However, an inherent limitation of our iMG analysis is the time delay between the date of blood collection and the date of analysis of iMG cells (after 14 days) due to the necessity of 14-day induction from blood monocytes. Further investigations should be conducted to clarify the impact of time delays in analysis of iMG cells.

The underlying biological mechanism shifting the expression patterns of iMG cells between depressive and manic states has not been clarified at present, while some internal and/or external factors are suggested to contribute to this shifting mechanism. Recent immunological studies have suggested that immune cell activities, including microglia, are modulated by the circadian clock system (33), methylation (34), and/or external stress (35), which may contribute to the activation patterns of microglia during clinical courses of bipolar disorder.

A previous study has shown that peripheral blood mononuclear cells from patients with rapid cycling bipolar disorder presented a different pattern of gene expression between manic and depressive states (36). In addition to the present study, this report also supports the premise that the cellular phenotype of microglia including M1/M2 state is different between depressive and manic states. Further studies are required to determine the clinical importance of these pilot findings.

## Conclusion

We introduced a novel translational approach focusing on bipolar disorder using the iMG technique. To our knowledge, this is the first report to indicate the importance of shifting microglial CD206 gene expression between depressive and manic states. This study is the first step toward understanding the contribution of microglia to the pathogenesis of bipolar disorders. Further studies are needed to dig up the microglial roles in bipolar disorder.

## Ethics Statement

The present study was conducted in accordance with the Declaration of Helsinki and was approved by the Ethics Committee of the Graduate School of Medical Sciences, Kyushu University.

## Author Contributions

All the authors contributed substantially to the scientific process leading up to the writing of the present manuscript. TK: the principal investigator of the present research; MO: the first author created the conception and design of the project and wrote the protocol. YH, TM, YM, TM-H, and AM: performed the clinical recruitment. MO, TK, YH, TM, YM, NS, and TM-H: performed the experiments and data analyses/interpretation. MO: wrote the first draft of the manuscript. TK, AM, YM, and SK: made critical revisions of the manuscript. All the authors approved this submission in its current form.

## Conflict of Interest Statement

The authors report no financial relationships with commercial interests.
