# Supplementary material for: Microglial CD206 Gene Has Potential as a State Marker of Bipolar Disorder
Source: Front Immunol. 2017 Jan 9;7:676. doi: 10.3389/fimmu.2016.00676 (PMC5220016; doi:10.3389/fimmu.2016.00676)
Supplement: Supplementary file 1 [file Data_Sheet_1.PDF]

Supplementary Materials

Materials and Methods

Clinical Recruitment

The present study was conducted in accordance with the Declaration of Helsinki and was approved by the Ethics Committee of the Graduate School of Medical Sciences, Kyushu University. We recruited three adult patients who were diagnosed with bipolar disorder I (rapid cycling type) based on the DSM-5 (**Table S1**). All participants provided written informed consent, after which we collected blood samples and administered several psychiatric assessments.

Case 1

The case of a 56 year-old man undergoing a long-term hospitalization in a closed psychiatric ward. In order to better understand his manic-depressive state, he consented to wear a wrist-band-type actigraph "Life Microscope (Hitachi Ltd., Japan)", which can measure 24-hour physical activity for a timeframe of more than one week on a single battery charge (37, 38). We collected peripheral blood during both manic and depressive phases shown at the arrows, respectively (**Figure 1**).

Case 2

The case of a 69 year-old man undergoing a short-term hospitalization in an open psychiatric ward.

Case 3

The case of a 54 year-old woman undergoing a long-term hospitalization in a closed psychiatric ward.

Induction of induced microglia-like (iMG) cells from human peripheral blood

The detailed methods of producing iMG cells were previously reported (13, 14). Briefly, peripheral blood was collected using a heparinized tube from patients with rapid cycling bipolar disorder. Peripheral blood mononuclear cells (PBMCs) were isolated by Histopaque-1077 (Sigma Chemical Co., St. Louis, MO, USA) density gradient centrifugation. PBMCs were resuspended with

RPMI-1640 (Nacalai Tesque, Kyoto, Japan), 10% heat-inactivated fetal bovine serum (FBS; Japan Bio Serum, Hiroshima, Japan), and 1% antibiotic/antimycotic (Invitrogen, Carlsbad, CA, USA). PBMCs were plated onto culture chambers at a density of 4 x 10^5 cells/ml and cultured overnight in standard culture conditions (37 °C, 5% CO_2). After overnight incubation, culture supernatant and non-adherent cells were removed. Adherent cells (monocytes) were cultured with RPMI-1640 Glutamax (Invitrogen) supplemented with 1% antibiotic/antimycotic and recombinant human GM-CSF (10 ng/ml; R&D Systems, Minneapolis, MN, USA) and recombinant human IL-34 (100 ng/ml; R&D Systems) for 14 days to develop the iMG cells (13, 14).

Quantitative real time-polymerase chain reaction (qRT-PCR)

To assess gene expression patterns in iMG cells, we performed qRT-PCR using a LightCycler 480 system (Roche Diagnostics, Mannheim, Germany). The iMG cells were washed, and total RNA was extracted using a High Pure RNA Isolation kit (Roche Diagnostics) according to the manufacturer's protocol, and subjected to cDNA synthesis using a Transcriptor First Strand cDNA Synthesis kit (Roche Diagnostics). qRT-PCR for BDNF, CCL-18, CD23, CD45, CD80, CD206, CD209, HLA-DR, IL-1β, IL-10, IL-23, and TNF-α was performed using their respective primers (**Table S2**). Beta 2-microglobulin of the Universal ProbeLibrary (Roche Diagnostics) Human beta-2-microglobulin (B2M) was used as a housekeeping control gene.

Statistical analyses

Statistical differences between groups (depressive state versus manic state) were analyzed by Student's *t*-test (two-tailed). All statistical analyses were performed using GraphPad Prism 6 (GraphPad Software, Inc., La Jolla, CA, USA). $P < 0.05$ was considered statistically significant.

Supplemental Data

Table S1. Demographic data of three patients with rapid cycling bipolar disorder

	Case 1		Case 2		Case 3	
**Sex**	male		male		female	
**Age**	56		69		54	
**Age of onset**	25		20		27	
**Duration of manic-depressive cycling**	45-55 days		40-60 days		60-100 days	
**State at the date of blood collection**	**Depressive**	**Manic**	**Depressive**	**Manic**	**Depressive**	**Manic**
**YMRS score**	**0**	**29**	**2**	**27**	**2**	**35**
**HAM-D score**	**17**	**8**	**11**	**0**	**25**	**14**
Depressed mood	3+	-	2+	-	4+	-

Loss of motivation/activity	3+	-	1+	-	4+	-
Insomnia	1+	2+	2+	-	1+	2+
Suicidal ideation	-	-	-	-	2+	-

Table S2. qRT-PCR primer sequences in the present study.

Primer	Sequences
CD45 (F)	5’-AGTCAAAGTTATTGTTATGCTGACAGA-3’
CD45 (R)	5’-TGCTTTCCTTCTCCCCAGTA-3’
CD80 (F)	5’-GAAGCAAGGGGCTGAAAAG-3’
CD80 (R)	5’-GGAAGTTCCCAGAAGAGGTCA-3’
HLA-DR (F)	5’-CCCAGGGAAGACCACCTTT-3’
HLA-DR (R)	5’-CACCCTGCAGTCGTAAACGT-3’
TNF-〈 (F)	5’-CAGCCTCTTCTCCTTCCTGAT-3’
TNF-〈 (R)	5’-GCCAGAGGGCTFATTAGAGA-3’
IL-1® (F)	5’-TACCTGTCCTGCGTGTTGAA-3’
IL-1® (R)	5’-TCTTTGGGTAATTTTTGGGATCT-3’
IL-23 (F)	5’-AGCTTCATGCCTCCCTACTG-3’
IL-23 (R)	5’-CTGCTGAGTCTCCCAGTGGT-3’

CD206 (F)	5’-CACCATCGAGGAATTGGACT-3’
CD206 (R)	5’-ACAATTCGTCATTTGGCTCA-3’
CD209 (F)	5’-AGCTGACCTGGCTGAAGG-3’
CD209 (R)	5’-GTTTCCTTGGAAGAATGTCCA-3’
CD23 (F)	5’-ACAGGAACTTGGAACAAGCAG-3’
CD23 (R)	5’-CCAGCAGCACGATCTGAGT-3’
BDNF (F)	5’-GTAACGGCGGCAGACAAA-3’
BDNF (R)	5’-GACCTTTTCAAGGACTGTGACC-3’
IL-10 (F)	5’-GATGCCTTCAGCAGAGTGAA-3’
IL-10 (R)	5’-GCAACCCAGGTAACCCTTAAA-3’
CCL18 (F)	5’-ATGGCCCTCTGCTCCTGT-3’
CCL18 (R)	5’-AATCTGCCAGGAGGTATAGACG-3’
